# Novel missense mutation in the *FH* gene in familial renal cell cancer patients lacking cutaneous leiomyomas

**DOI:** 10.1186/1756-0500-7-203

**Published:** 2014-03-31

**Authors:** Masaomi Kuwada, Yoshitomo Chihara, Yi Lou, Kazumasa Torimoto, Yoriaki Kagebayashi, Kenji Tamura, Taro Shuin, Kiyohide Fujimoto, Hiroki Kuniyasu, Shoji Samma

**Affiliations:** 1 Department of Urology, Nara Medical University, 840, Shijyo-cho, Kashihara, Japan; 2 Department of Molecular Pathology, Nara Medical University, 840, Shijyo-cho, Kashihara, Japan; 3 Department of Urology, Nara Prefectural Nara Hospital, 1-30-1, Hiramatsu, Nara, Japan; 4 Department of Urology, Kochi Medical School, Nankoku, Kochi, Japan

**Keywords:** Familial renal cell cancer, Papillary renal cell cancer, Fumarate hydratase, Missense mutation

## Abstract

**Background:**

Hereditary leiomyomatosis and renal cell cancer (HLRCC) is a rare tumor predisposition syndrome characterized by cutaneous and uterine leiomyomas and papillary type 2 renal cell cancer. Germline mutation of the fumarate hydratase (*FH*) gene is known to be associated with HLRCC.

**Case presentation:**

We describe a 64-year-old father and his 39-year-old son with HLRCC who developed papillary type 2 RCCs lacking cutaneous leiomyomas at any site. A common missense mutation in the *FH* gene, (c.1021G > A, p.D341N) in exon 7, was detected in the 2 cases. Functional prediction with the bioinformatics programs, SIFT and Polyphen-2, reported “damaging (SIFT score 0.00)” and “probably damaging (PSIC score 1.621)” values, respectively. In 162 healthy individuals, there were no cases of a G transition to any base. Finally, (c.1021G > A) in exon 7, was identified as a point mutation.

**Conclusion:**

We report a family with HLRCC in which a novel missense mutation was detected. A familial papillary type 2 renal cancer should be considered HLRCC unless typical cutaneous leiomyomas do not occur.

## Background

Hereditary leiomyomatosis and renal cell cancer (HLRCC: OMIM 605839) is an inherited autosomal dominant cancer syndrome characterized by cutaneous leiomyoma, uterine leiomyoma, and/or a single renal tumor [1]. Cutaneous leiomyomas are the commonest clinical feature, arising in over 80% of HLRCC patients, and RCC is found in 20-35% of them with an early onset often at 30-50 years of age [2]. The renal cell cancers in this syndrome are mostly papillary type 2 RCC, which are often solitary and unilateral, and they are more likely to be aggressive. HLRCC is caused by heterozygous germline mutations in the fumarate hydratase (*FH*) gene, which is located at 1q42.3-43 [3]. The *FH* gene codes for fumarate hydratase, which is an enzyme that catalyzes the conversion of fumarate to malate in the Krebs cycle. Mutations of the *FH* gene, including missense, frameshift, and complete or partial deletions, have been detected in 90% of HLRCC families, suggesting that the *FH* gene has the characteristics of a tumor suppressor gene. HLRCC is comparatively rare, and so far, 180 HLRCC families have been identified worldwide [4]. The *FH* mutation database provides a total of 155 variants, as of the last up-date on August 17, 2011 [5]. Here, we present a HLRCC family with arising papillary type 2 RCCs, in which a novel missense germline mutation in the *FH* gene was detected.

## Case presentation

### Case 1

**Figure 1:**
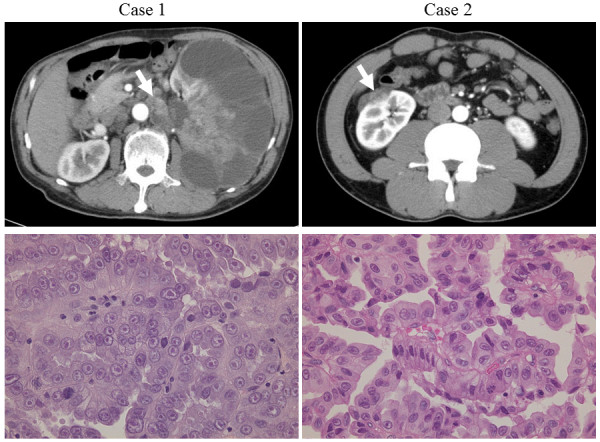
**CT imaging and pathology of renal cancers arising in a family with HLRCC.** Case 1: A large renal cell cancer of the left kidney and para-aortic lymph node swelling (arrow). Case 2: Right renal mass appearing predominantly less enhanced compared with the normal renal parenchyma (arrow). Pathological findings of both cases showed papillary architectures, large cells, spherical nuclei, and varying degrees of nuclear pseudostratification. The cytoplasm was voluminous and eosinophilic. (H&E, × 400).

A 64-year-old man had anorexia and body weight loss of 10 kg for 3 months before referral. Contrast enhanced computed tomography (CECT) revealed a huge mass arising on his left kidney (Figure 1), and swelling in his pulmonary and para-aorta lymph nodes. On admission, Karnofsky performance status was 80, blood chemical analysis revealed Hemoglobin (Hb): 12.1 g/dL, Lactate dehydrogenase (LDH): 239 IU/L, Corrected serum calcium (Ca): 9.1 mg/dL. This case was categorized into poor risk according to MSKCC (Memorial Sloan-Kettering Cancer Center) criteria [6]. Cytoreductive nephrectomy was performed in September 2007, and a pathological diagnosis indicated papillary renal cell carcinoma type 2 with adjacent lymph-node involvement (pT4N2M1). The patient developed systemic lymph node and bone metastasis after surgery. Although he was treated with interferon-α, interleukin-2, sunitinib, and palliative radiation therapy, he died 17 months after surgery.

### Case 2

A 39-year-old man, who was the eldest son of the patient in Case 1, did not have any symptoms but asked for extensive testing. CECT revealed a right renal tumor 3 cm in diameter (Figure 1). Laparoscopic radical nephrectomy was performed in November 2008, and the tumor was determined to be pathological papillary renal cell carcinoma type 2 (pT1aN0M0). Systemic lymph node and lung metastasis arose 11 months after nephrectomy. At that point, the cases show no abnormal hematological findings, was categorized into favourable risk according to MSKCC criteria. He was treated with interferon-α kept stable disease for 12 months. However, metastasis also arose in the fourth lumbar vertebra at 23 months after nephrectomy. He was treated with temsirolimus. Finally, he died 50 months after surgery.

**Figure 2:**
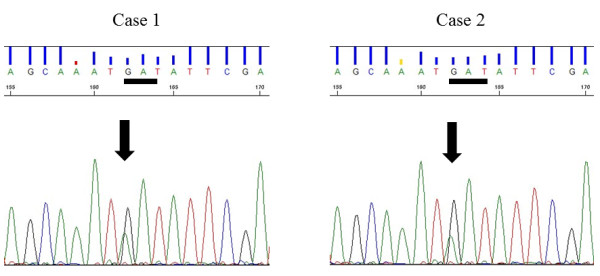
**Partial sequence of the *FH *gene in exon 7.** Heterozygous mutations (c.1021G > A) were found in both Case 1 and 2.

Written informed consent was obtained from both patients before their deaths, for germline mutation analysis of the von Hippel-Lindau *(VHL)* and *FH* gene, and the study was approved by the Medical Ethic Committee of Nara Prefectural Nara Hospital. DNA was extracted from their peripheral blood leukocytes (PBLs), and was directly sequenced to include all coding exons of *VHL* and *FH* as described previously [7,8]. No mutation was detected in *VHL*. However, the analysis revealed a single missense mutation (c.1021G > A, p.D341N) in exon 7 of *FH*, which was common to both cases (Figure 2). This missense mutation has not been reported.

### Genetic findings

**Figure 3:**
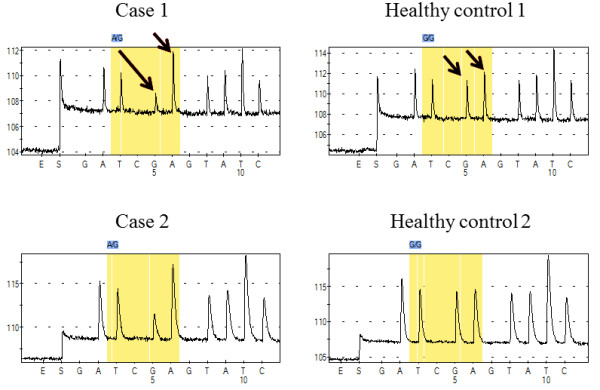
**Pyrograms of genotyping analysis.** The peak at the fifth base position, “G” in the HLRCC cases, decreased. Subsequently the peak of “A” increased, relatively. Wild type showed the same peak between G and A. This genotyping revealed that the HLRCC cases’ genotypes were G/A in this position, which was G in the wild type.

*In silico*, SIFT (Sorting Intolerant from Tolerant [9]) analysis provided a SIFT score of (p.D341N) for “0.00”. This score indicates that the mutations is “damaging”. Similarly, Polyphen-2 (Polymorphism Phenotyping-2 [10]) provided a PSIC score of “1.621”, which indicates that the mutation is “possibly damaging”. We performed genotyping analysis of the missense mutation using 162 healthy blood samples. Because (c.1021G > A) is a novel *FH* mutation, we should exclude the possibility that this mutation might be a novel single nucleotide polymorphism (SNP), which is defined as a minor allelic frequency of more than 1%. Both the HLRCC cases showed (c.1021G > A). There was no case of a G transition to any base among 162 healthy individuals (Figure 3). Finally, (c.1021G > A) in exon 7 was identified as a point mutation.

## Conclusion

Hereditary renal cell cancer comprises an estimated 3-5% of RCCs, and to date, 10 HRC syndromes have been described [11]. Clear cell RCC is the most common type, accounting for 70-80% of all RCCs, and *VHL* inactivation is very common in both sporadic clear cell RCC and the hereditary cancer syndrome known as von Hippel-Lindau disease [12]. Papillary RCC accounts for 10-15% of all RCCs. Papillary RCCs are divided into type 1 and type 2, with the object of histopathological findings. The *MET* gene mutation occurs in both hereditary and sporadic papillary type 1 RCCs, whereas the *FH* gene mutation is detected only in hereditary papillary type 2 RCC as a germline mutation [8].

The *FH* gene encodes fumarate hydratase, which is an enzyme of the Krebs cycle that catalyzes the conversion of fumarate to malate. Thus, FH deficiencies are thought to result in chronic accumulation of fumarate, and lead to competitive inhibition of hypoxia induced factor (HIF) prolyl hydroxylase (HPH). Under normoxic conditions, hydroxylated HIF is recognized by the VHL complex and targeted for ubiqutin-mediated degradation. When HPH is inhibited, HIF remains unhydroxylated and avoids degradation. Then, accumulated HIF consequently upregulates HIF target genes such as VEGF, GLUT1, PDGF, and TGFα, which facilitate tumor growth [13]. Thus, the molecular pathway for tumor growth in FH deficiencies closely resembles VHL deficiency in clear RCCs. However, no association has been verified between the renal cancer phenotype and the type or location of the *FH* mutation or FH enzyme activity [14]. Indeed, to address the FH deficiencies in the tumor tissues, we performed immunohistochemistry of FH and HIF1. We did not find any difference in FH and HIF1 expression between HLRCC cases and clear RCC or normal kidney tissues (data not shown).

The most common type of *FH* mutation is missense (57%), followed by frameshift and nonsense (27%), and diverse deletions, splice site and duplications [15]. A total of 73 missense mutations have been reported, spanning exons 2 to 10. Mutations in exon 7 are the most common, and account for 23 of the total reported mutations. Our cases showed a common missense mutation (c.1021G > A, p.D341N), and the same substitution (c.1021G > T, p.D341Y) was also reported [16]. From the genotyping results, these substitutions were defined as missense mutations. Several missense mutations around (c.1021G) were reported i.e. (c.1002 T > G), (c.1004 T > C), and (c.1020 T > A). These loci might be a hot spot for *FH* mutations. A very small number of these mutations have known changes in FH enzymatic activity, although most of them have SIFT scores of “0.00”.

HLRCC-associated renal cancers are early onset and very aggressive, and most reported patients died within 5 years of diagnosis [17]. Radical surgery may be beneficial in the early stages, although the optimal management for HLRCC has not been established. A potential promising results for locally advanced and metastatic papillary RCC have been reported with mammalian target of rapamycin (mTOR) inhibitors, temsirolimus. Exploratory subgroup analysis of 3-arm phase III trial comparing temsirolimus, interferon-α or both including 55 papillary RCCs revealed that temsirolimus group had prolonged overall survival and progression-free survival compared to patients treated with interferon-α [18]. Another potential agent might be erlotinib, an oral epidermal growth factor receptor (EGFR) tyrosine kinase inhibitor. A multicenter phase IItrial of erlotinib in patients with locally advanced and metastatic papillary RCCs revealed an overall response rate of 11% with an additional 53% experiencing stable disease [19]. Unfortunately these data are not specific for HLRCC. A crinical trial evaluating the combined effect of bevacizumab and erlotinib in patients with advanced HLRCC is currently under way [20].

Smit et al. [21] advocated the proposed criteria for the clinical diagnosis of HLRCC as follows. Major criterion: multiple cutaneous pioleiomyomas confirmed histopathologically. Minor criteria: 1) surgical treatment for severely symptomatic uterine leiomyomas before age 40, 2) type 2 papillary renal cell carcinoma before age 40, and 3) a first-degree family member who meets one of the above-mentioned criteria. The diagnosis is likely when a proband meets the major criterion, and HLRCC may be suspected when a proband meets at least 2 minor criteria. However, in our cases, no cutaneous leiomyomas were found, but the first-degree family developed early onset papillary type 2 RCC with the same pathological features as the father, and this should be primarily considered as HLRCC.

To our best knowledge, a novel missense mutation of the *FH* gene was confirmed by genotyping and *in silico* analysis. Papillary type 2 RCCs, which have a familial occurrence, should be used for HLRCC identification, if no cutaneous leiomyomas develop. When a germline mutation is identified, all family members should be offered appropriate counseling and genetic testing.

## Material and methods

### DNA extraction

Peripheral blood leukocytes (PBLs) from 162 healthy cohorts were used for consecutive genetic analysis. These samples were collected by following protocols that were approved by the Medical Ethics Committee of Nara Medical University. Written informed consent was obtained from all participants. DNA was extracted according to standard procedures.

### Functional prediction of FH mutation in silico

The impact of the (p.D341N) missense mutation on protein function was assessed with SIFT and PolyPhen-2. Both SIFT and PolyPhen-2 are online prediction tools that predict the degree of impact of an amino acid substitution on the structure and function of a human protein. SIFT provides a SIFT Score in which a value of less than 0.05 indicates that the amino acid substitution is predicted to be “Damaging”. PolyPhen-2 provides a position-specific, independent count (PSIC) score difference, which was assigned using the categories “probably damaging” (2.00 or more), “possibly damaging” (1.40-1.90), “potentially damaging” (1.20-1.50), “borderline” (1.00-1.20) and “benign” (0.00-0.90).

### Genotyping by pyrosequencing

One hundred and sixty-two healthy control samples were Genotyped at nt 1021 on *FH* by pyrosequencing (PSQ) using PyroMark Q96 (Qiagen), according to the manufacture’s protocol. To enable single-strand preparation, the reverse primer was 5′-biotinylated. Reaction volumes of 30 μl contained 5× GoTaq buffer, 1.5 units of GoTaq Hot Start Polymerase (Promega), 1 μM of primers, and 500 nM of dNTPs. PCR conditions were as follows: 95°C for 3 min; 45 cycles of 95°C for 30 s, 58°C for 30 s, and 72°C for 30 s; and a final extension step at 72°C for 4 min. PCR primer sequences were: 5′-TTCTGTTTCACTTGCTAATGGTAGA-3′ (forward), and 5′-GGACCTAGTCAAGTTTTAGCTCCA-3′ (reverse), respectively. The sequencing primer sequence was 5′-AGTCTGATGAAGATAGCAA-3′. We designed this assay so that if c.1021G > A was an unreported SNP, it could detect all nucleotide variations. Sequences to analyze were ATG/NATATTC.

## Consent

Written informed consent was obtained from these patients’ next of kin for publication of this case report and any accompanying images. A copy of the written consent is available for review by the Editor-in-Chief of this journal.

## Competing interests

The authors declare that they have no competing interests.

## Authors’ contributions

MK participated in the genetic analysis and drafted the manuscript. YC conceived the study idea, and participated in its design and coordination and in writing of the manuscript. YL participated in genetic analysis. KT and YK managed the patients. KT and TS participated in VHL mutation analysis. KF collected blood samples from healthy subjects at Nara Medical University, Kashihara, Japan and gained ethics committee approval to enroll this study. HK managed histopathological findings of the patients. SS managed the patients, gained ethics committee approval for genetic analysis of this study at Nara Prefectural Nara hospital, Nara, Japan, and participated in writing of the manuscript. All authors read and approved the final manuscript.
